# Influence of systematic standard and Nordic walking training on exercise tolerance and body weight components in women over 55 years of age

**DOI:** 10.3389/fspor.2025.1568491

**Published:** 2025-03-10

**Authors:** Vera Knappova, Kopeć Dorota, Witkowska Anna, Gabriela Kavalirova, Nowak Zbigniew, Tomasz Gabryś, Nowak-Lis Agata

**Affiliations:** ^1^ Department of Physical Education and Sport Science, Faculty of Pedagogy, University of West Bohemia, Pilsen, Czechia; ^2^ Department of Physiotherapy, Jerzy Kukuczka Academy of Physical Education, Katowice, Poland

**Keywords:** March, Nordic walking, body mass composition, exercise tolerance, physical activity

## Abstract

**Background:**

The most physiological form of movement of human body is walking. The aim of the study was to assess the changes before and after workout programme in body mass components and exercise tolerance in women above 55 years of age, both in standard walking (March training) and walking with poles (Nordic Walking).

**Material and methods:**

77 (55–64 years) women were divided into two groups: I - 37 women, participating in the marching training. II - 40 women participating in the Nordic Walking training. CPET, and body composition analysis were performed in each of the participants before and after workout program.

**Results:**

Significant changes in VO2peak, both in standard walking group and Nordic walking, distance and test duration were observed. The evaluation of body composition Standard and Nordic Walking groups concerned statistically significant changes in the same indicators at the same level of significance: fat content (%), water content inside and outside the cell (%). In Nordic walking group there where some significant correlations between the changes in body fat (%), body weight, visceral obesity and fitness scores, changes in metabolic cost associated with the exercise test, changes in body fat (%) content and increase in the duration of the exercise test, as well as changes in body fat (%) content and increase in the distance of the test.

## Background

Aging is a physiological process that affects the functioning of most organs and systems. The most characteristic features are a decrease in physical and mental performance, unfavorable changes in the cardiovascular system, decreases in muscle and bone mass, and disturbances in balance and motor coordination. This leads to functional problems and a loss of physical independence. Additionally, numerous concomitant chronic diseases overlap to the detriment of the degenerative process in the elderly (1). Achieving a long life in health, fitness and independence, while delaying the involutionary process, should be sought in pro-health and preventive activities, consisting in an appropriate lifestyle. Regular physical activity, especially in contact with nature, plays a huge role in improving the physical condition and, consequently, mental health (2, 3). Exercise carried out in a natural environment, in the open air or in urban green spaces is better than exercise in closed rooms (4). The most physiological form of movement of human body is walking. A modification of this form of activity is Nordic walking (NW). Using poles during walking engage the muscles of the upper body, which are passive during normal walking. Normal walking and NW are recommended in all age groups (especially the elderly), including people with low physical capacity, overweight or obese people, those with diabetes, chronic obstructive pulmonary disease, Parkinson's disease, or peripheral vascular diseases, pregnant women, and patients at risk (e.g., after a heart attack) in the convalescence phase (5–9).

NW requires a higher expenditure of energy at a given speed compared to normal walking. Nordic walking ensures balance and correct posture, prevents stumbling and falls as well as improves functional fitness, and lowers blood pressure (10, 11). Even if this kind of training is quite popular this days, the number of reports with recommendations related to it for women during or after the menopause (>55 years of age) is small. Most of them are general in nature, without detailed clinical analyses (11, 12). Monitoring of body composition is of significant importance in the assessment of health status, the level of functional capacity, and the course of the aging process (13).

Adverse changes in one or more of its elements increase the risk of age-related diseases (14). Thus, monitoring of body composition has become crucial in assessing the nutritional status of the elderly and middle-aged. One of the most popular methods of assessing body composition is bioelectric impedance analysis (BIA) due to its combination of cost effectiveness, ease of use and portability (15). The accuracy of such a study is quite high, in the range of 3.5%–5% (16). It is difficult to state conclusively which of this two mentioned forms of walking would be the most beneficial in terms of changes in body weight composition, exercise tolerance, and lipid profile for women who enter the period of slow aging after menopause.

Therefore, the aim of the study was to analyze the changes occurring before and after training in the components of body mass and exercise tolerance under in women who systematically use various forms of walking training – ordinary walking training and walking with poles (Nordic walking).

The following research questions were formulated:

1. Which of the two types of systematic and structured walking training (walking without or with poles) leads to greater changes in body weight in mature women (>55 years of age)?
2. Do both forms of training improve exercise tolerance in a similar way?
3. Is there a relationship between the change in the level of exercise tolerance in women (in the analyzed groups) and the change in the composition of their body weight?

## Material and methods

Presented studies are part of research that showed differences between Nordic walking and standard walking at the group of women over 55 years old.

The study was conducted as a randomized controlled longitudinal study with 2 parallel training groups and was considered an exploratory study. The study was approved by the Bioethical Committee (No. 7/2011) and was in line with the standards set out in the Helsinki Declaration.

All patients were informed of the nature and purpose of the study and provided written informed consent prior to enrollment in the study. Participants could withdraw from the study at any time.

### Participants

86 women aged >55 participated in the first stage of the study, taking part in regular recreational activities. The aim of this stage was to eliminate the risk of health or life caused by the presence of disease or insufficient physical capacity. Therefore, each of the examined women was thoroughly diagnosed in a hospital setting (blood laboratory tests, exercise tests on a treadmill, echocardiography of the heart).

Inclusion criteria for the study were: postmenopausal women >55 years old, LVEF ≥50%, normal values of blood pressure or pharmacologically controlled mild hypertension (140–159/90–99 mmHg), stable coronary artery disease. Exclusion criteria: COPD (stage II, III, IV), recent myocardial infarction (<3 months), confirmed symptoms of heart failure, musculoskeletal dysfunction impeding walking, unregulated blood glucose levels, left ventricular ejection fraction <50%, unregulated hypertension grade II or III hypertension, history of acute thrombosis or embolism.

Taking into account the adopted inclusion and exclusion criteria, 77 women were qualified for the second part of the research project. Body weight composition was assessed in each of the women.

**Figure 1:**
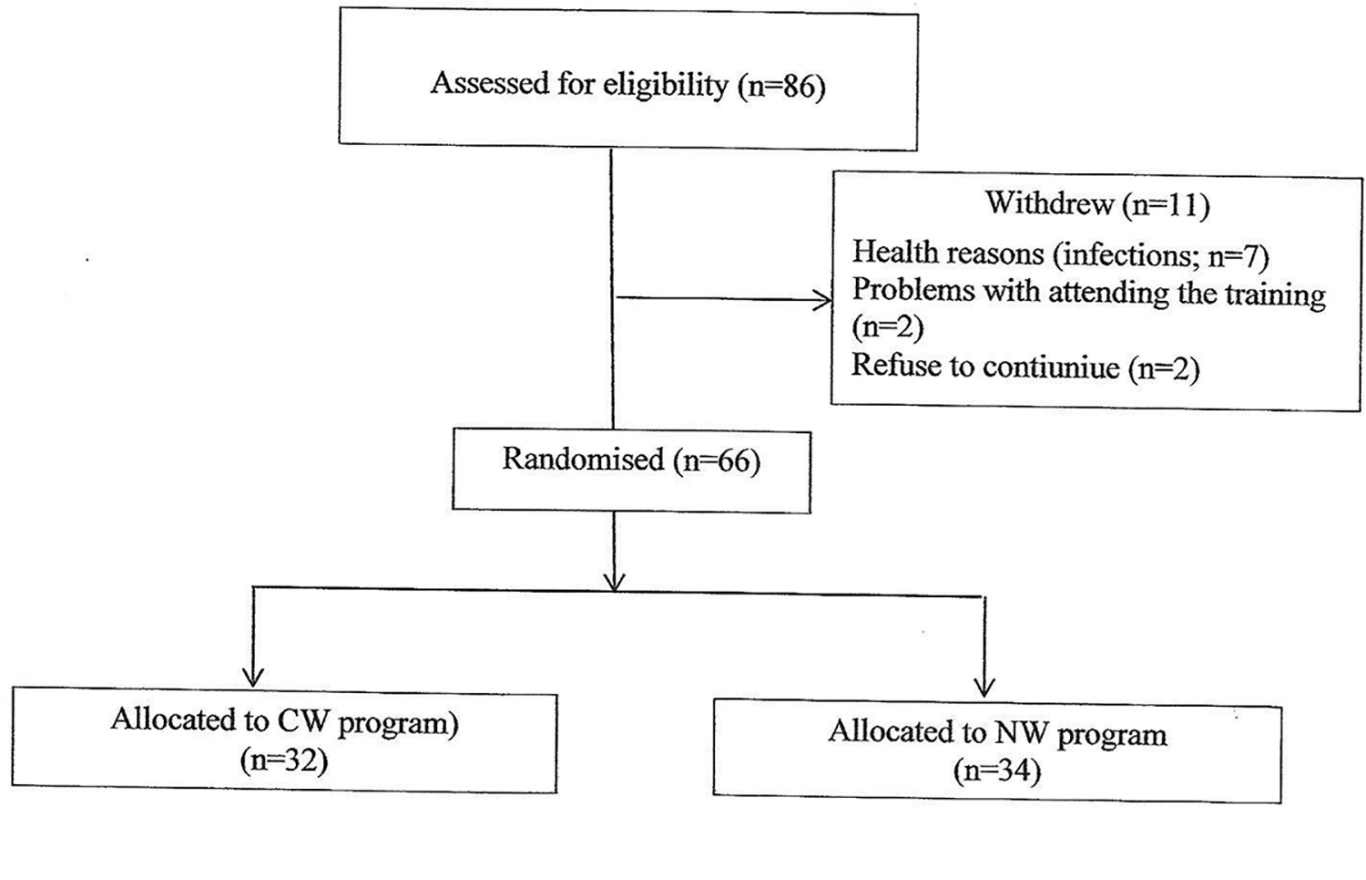
Flow diagram.

Participants were randomly assigned to the CW (conventional) or NW (Nordic walking) group using permuted block randomization [Microsoft Excel 2019 (version 16.0); Microsoft Corp., Redmond, WA]. After starting training programme, several women (*n* = 11) withdrew from the study due to health conditions (infections, *n* = 7) or other reasons (problems participating in the training program, *n* = 2; refusal to continue, *n* = 2). 66 women were included in the final analysis (Figure 1).

### Methods

The tests were carried out on all participants at the beginning (pre-test) and after 12 weeks (*post test*). The spiroergometric submaximal treadmill stress test was performed with a METAMAX 3B CORTEX gas analyzer (CORTEX Biophysik GmbH, Leipzig, Germany) according to Bruce's protocol [seven 3-min levels with increasing speed and incline: starting at 1.7 mph from 10% gradient (upward slope) to a maximum of 5.5 mph with a 20% gradient]. Drugs were not discontinued prior to the study. The following parameters were assessed during the test: resting heart rate (HRrest) and peak heart rate (HR peak) [bpm], systolic blood pressure at rest (SBPrest) and peak systolic blood pressure (SBPpeak) [mmHg], diastolic blood pressure at rest (DBPrest) and peak diastolic blood pressure (DBPpeak) [mmHg], metabolic equivalents (METs; ml O_2_/kg/min), peak oxygen consumption [VO_2_ peak] achieved during the submaximal test [ml/kg/min], distance [m], test duration [min]. Reasons for finishing the test included physiological: submaximal heart rate reached, which was determined by the formula: (208−0.7 × age) × 0.85 or fatigue and pathological stenocardial pain, deviation in the ST–T segment, arrhythmia and conduction disorders, and increases in blood pressure >250/120 mmHg.

The body mass composition was measured using the InBody 720 device. The following indicators were assessed:

Fitness score – based on the replacement of the muscle and fat fractions in relation to body weight:

≤70 – weak, obese type
70–90 – normal, healthy type
≥90 – athletic type
Visceral fat area (VFA) [cm^2^]
Body mass [kg]
Skeletal muscle mass (SMM) [kg]
Body fat mass (BFM) [kg]
Percent body fat (PBF) [%]
The risk limits are set at 10%–20% for men, 18%–28% for women
Extracellular water (ECW) [%]
Intracellular water (ICW) [%]
Total body water (TBW) [kg]

Additionally, the level of left ventricular ejection fraction (LVEF) was assessed using echocardiography (ultrasound ALT HDI 3000; Philips, Best, Netherlands). The tests were performed by a cardiologist using 1-dimensional M-mode and 2-dimensional projection in accordance with the recommendations of the American Society of Echocardiography.

Conventional walking and NW were performed 3 times a week for 12 weeks (Table 1). Each training session was held outdoors on an unpaved ground. The effort intensity was estimated using Borg scale: (scale 6–20). All participants were familiar with the scale (perceived exertion rating 11–13). The entire training was supervised by the same physiotherapists who were also NW instructors. All women were taught the correct CW and NW techniques during the first training. All participants were asked not to modify their diet and not to undertake additional physical activity during the training programme. All training sessions, both the classic walking training and Nordic walking, were conducted under the same environmental conditions (a forest park in the vicinity of Katowice, on flat terrain without inclines) as well as at the same time of day (between 2:00 PM and 4:00 PM).

**Table 1:** Walking training programs.

Type of exercises	Frequency	Duration	Intensity
Conventional walking	3 days/week	60 min/day (10-min warmup, 40-min main part, and 10-min cooldown)	Moderate 11–13 rating of perceived exertion
Nordic walking			

### Statistical analysis

Results are expressed as mean and standard deviation (SD) of the parameters. The normality of the distribution was verified with the Shapiro test, and homogeneity of variance was verified by the Brown–Forsythe test. Age, body weight were compared with the *t*-test or the Mann–Whitney *U*-test. It was used repeated-measures analysis of variance with 95% confidence intervals to determine the effects of the NW and CW training programs on aerobic capacity, body mass, and BMI with the pre-exercise and post-exercise measurements as factors. The *post hoc* Tukey test was performed when a significant main effect was detected. To avoid the likelihood of type I error, Bonferroni correction was applied. A high value of power analysis (above 0.8) means that the sample size was sufficient. For imputing missing data (results of posttests) of 8 participants who withdrew from the study after starting the intervention, the hot-deck imputation method was applied. The level of significance was set to *P* ≤ .05. The statistical analysis was performed using STATISTICA software v.10.

## Results

The results were analyzed with the intention-to-treat method (participants randomly allocated to one of the walking programs were included in the statistical analysis and analyzed according to the CW or NW group allocation (Figure 1). Participants Baseline Characteristics). The mean age of the CW group was 62.81 ± 5.25 years and body weight was 71.70 ± 13.81 kg, whereas the mean age of the NW group was 61.88 ± 4.87 years and body weight was 70.34 ± 11.97 kg. The differences in age and body weight were not statistically significant.

### Analysis of electrocardiographic exercise test results

After the end of the training program, both the walking group and the Nordic walking group showed a significant increase in exercise tolerance, manifested by an increase in peak heart rate, maximum systolic pressure, oxygen uptake, distance, MET energy expenditure, and an increase in the duration of the exercise test. The intergroup analysis concerning comparisons in terms of changes in the values of individual test values (delta) showed significant differences in the duration of the test, the distance covered and the maximum oxygen consumption (Table 2).

**Table 2:** Results of stress tests before (1) and after (2) training completion.

Variable	CWG			NWG		
	$\bar{x}$ ± SD	Δ	*p*	$\bar{x}$ ± SD	Δ	*p*
HRrest [beats/min] 1	82.43 ± 11.89	−1.90	0.098	79.67 ± 10.49	−0.97	0.103
HRrest [beats/min] 2	80.53 ± 10.53			78.70 ± 9.93		
ΔCWG vs. ΔNWG	0.533					
HRpeak [beats/min] 1	137.40 ± 17.48	5.25	0.015	137.35 ± 16.17	4.64	0.009
HRpeak [beats/min] 2	142.65 ± 14.66			142.00 ± 14.12		
ΔCWG vs. ΔNWG	0.881					
SBPrest [mmHg] 1	127.03 ± 14.69	−0.15	0.862	129.85 ± 19.52	−0.88	0.789
SBPrest [mmHg] 2	126.87 ± 10.93			128.97 ± 13.19		
ΔCWG vs. ΔNWG	0.563					
DBPrest [mmHg] 1	77.50 ± 10.93	−1.71	0.359	75.29 ± 10.44	0.88	0.443
DBPrest [mmHg] 2	75.78 ± 9.17			76.17 ± 7.98		
ΔCWG vs. ΔNWG	0.879					
SBPpeak [mmHg] 1	183.28 ± 28.16	3.90	0.032	180.00 ± 28.39	2.79	0.046
SBPpeak [mmHg] 2	187.18 ± 24.43			182.79 ± 22.40		
ΔCWG vs. ΔNWG	0.935					
DBPpeak [mmHg] 1	89.37 ± 16.79	3.43	0.013	85.14 ± 13.95	1.61	0.194
DBPpeak [mmHg] 2	92.81 ± 16.94			86.76 ± 12.42		
ΔCWG vs. ΔNWG	0.355					
MET 1	9.05 ± 2.05	0.46	0.019	8.82 ± 2.11	0.78	0.01
MET 2	9.51 ± 2.18			9.60 ± 2.09		
ΔCWG vs. ΔNWG	0.419					
VO_2peak_ 1 [ml/kg/min]	27.47 ± 7.09	2.33	0.002	26.61 ± 7.81	4.37	0.000
VO_2peak_ 2 [ml/kg/min]	29.80 ± 8.54			30.98 ± 10.31		
ΔCWG vs. ΔNWG	0.035					
Distance [m] 1	256.34 ± 97.91	39.34	0.000	247.83 ± 107.06	59.71	0.000
Distance [m] 2	295.68 ± 113.6			307.55 ± 128.58		
ΔCWG vs. ΔNWG	0.045					
Duration [min] 1	6.75 ± 2.09	0.63	0.001	6.47 ± 2.24	1.13	0.000
Duration [min] 2	7.38 ± 2.06			7.60 ± 2.32		
ΔCWG vs. ΔNWG	0.002					

All data are presented as mean ± standard deviation and the difference (Δ, delta).

CWG, conventional walking group; NWG, Nordic walking group; HRrest, heart rate at rest; SBPrest, systolic blood pressure at rest; DBPrest, diastolic blood pressure at rest; MET, metabolic equivalent; VO_2_ peak, peak oxygen consumption; HRpeak, peak heart rate; SBPpeak, peak systolic blood pressure; DBPpeak, diastolic blood pressure.

### Analysis of changes in BMI

After the end of the training program in both the walking group and the Nordic walking group, the result did not change. The intergroup analysis also showed no significant differences.

### Analysis of changes in body mass composition indices

In both analyzed training groups, significant changes in the percentage of adipose tissue, external and intracellular water were observed. The intergroup analysis of changes in the examined indicators did not show any significant differences (Table 3).

**Table 3:** Comparison of BMI values before (1) and after (2) the end of training.

Variable	CWG			NWG		
	$\bar{X}$ ± SD	Δ	*p*	$\bar{X}$ ± SD	Δ	*p*
BMI [kg/m^2^] 1	29.61 ± 3.88	−0.3	0.634	27.94 ± 5.00	−0.06	0.696
BMI [kg/m^2^] 2	29.30 ± 3.91			27.87 ± 5.02		
ΔCWG vs. ΔNWG	0.322					

All data are presented as mean ± standard deviation and the difference (Δ, delta).

CWG, conventional walking group; NWG, Nordic walking group; BMI, body mass index.

### Correlation of the level of exercise tolerance and body mass components

#### CWG – conventional walking group

There were no statistically significant correlations.

#### NWG – Nordic walking group

The results of the analysis show a significant positive relationship between the BMI index and the increase in the duration of the exercise test, metabolic cost and maximum oxygen consumption (Table 4).

**Table 4:** Comparison of body mass composition indices before (1) after (2) training completion.

Variable	CWG			NWG		
	$\bar{X}$ ± SD	Δ	*p*	$\bar{X}$ ± SD	Δ	*p*
Fitness score 1	68.61 ± 6.30	−1.21	0.581	68.20 ± 6.49	−0.93	0.702
Fitness score 2	67.40 ± 5.84			67.26 ± 6.68		
ΔCWG vs. ΔNWG	0.543					
VFA [cm^2]^ 1	136.04 ± 33.74	2.27	0.834	134.37 ± 25.90	10.47	0.360
VFA [cm^2^] 2	138.31 ± 24.50			144.85 ± 35.11		
ΔCWG vs. ΔNWG	0.121					
Body mass [kg] 1	71.70 ± 13.81	−1.47	0.759	70.34 ± 11.97	1.90	0.689
Body mass [kg] 2	70.23 ± 12.27			72.24 ± 13.78		
ΔCWG vs. ΔNWG	0.741					
SMM [kg] 1	24.21 ± 3.06	−0.86	0.476	23.62 ± 3.16	0.39	0.734
SMM [kg] 2	23.35 ± 3.43			24.01 ± 3.14		
ΔCWG vs. ΔNWG	0.532					
BFM [kg] 1	27.20 ± 9.882	0	0.999	26.85 ± 8.13	1.34	0.688
BFM [kg] 2	27.20 ± 7.815			28.20 ± 9.94		
ΔCWG vs. ΔNWG	0.489					
PBF [%] 1	0.98 ± 9.88	129.28	0.001	0.98 ± 0.04	133.29	0.001
PBF [%] 2	130.26 ± 17.48			134.27 ± 27.53		
ΔCWG vs. ΔNWG	0.989					
ECW [%] 1	20.09 ± 2.35	−7.91	0.001	19.64 ± 2.42	−7.23	0.001
ECW [%] 2	12.18 ± 1.62			12.40 ± 1.39		
ΔCWG vs. ΔNWG	0.989					
ICW [%] 1	12.61 ± 1.41	6.81	0.001	12.34 ± 1.48	7.60	0.001
ICW. [%] 2	19.43 ± 2.62			19.94 ± 2.14		
ΔCWG vs. ΔNWG	0.989					
TBW [kg] 1	32.70 ± 3.75	−1.09	0.460	31.987 ± 3.89	0.36	0.798
TBW [kg] 2	31.61 ± 4.23			32.350 ± 3.79		
ΔCWG vs. ΔNWG	0.345					

All data are presented as mean ± standard deviation and the difference (Δ, delta).

CWG, conventional walking group; NWG, Nordic walking group; VFA, visceral fat area; BFM, body fat mass; SMM, skeletal muscle mass; BFM, body fat mass; PBF, percent body fat; ICW, intracellular water; ECW, extracellular water; TBW, total body water.

The results of the analysis show a significant correlation between changes in body fat (%), body fat mass, body weight, visceral obesity and fitness score and changes in the metabolic cost associated with the exercise test, changes body fat (%) and the increase in the duration of the exercise test, as well as changes body fat( %) content and the increase of distance during the test (Tables 5–8).

**Table 5:** Correlation of BMI values with the results of the exercise test.

Variable	Δ HRrest [beats/min]	Δ SBPrest [mmHg]	Δ DBPrest [mmHg]	Δ Duration [min]	Δ MET	Δ VO_2peak_ [ml/kg/min]	Δ Distance [m]	Δ HRpeak [beats/min]	Δ SBPpeak [mmHg]	Δ DBPpeak [mmHg]
ΔBMI [kg/m^2^]	−0.100	−0.183	0.067	−0.147	−0.273	0.056	−0.057	−0.021	−0.166	0.089

BMI, body mass index; HRrest, heart rate at rest; SBPrest, systolic blood pressure at rest; DBPrest, diastolic blood pressure at rest; MET, metabolic equivalent; VO_2_ peak, peak oxygen consumption; HRpeak, peak heart rate; SBPpeak, peak systolic blood pressure; DBPpeak, diastolic blood pressure.

**Table 6:** Correlation of the values of body mass composition indices with the results of the exercise test.

Variable	Δ HRrest [beats/min]	Δ SBPrest [mmHg]	Δ DBPrest [mmHg]	Δ Duration [min]	Δ MET	Δ VO_2peak_ [ml/kg/min]	Δ Distance [m]	Δ HRpeak [beats/min]	Δ SBPpeak [mmHg]	Δ DBPpeak [mmHg]
ΔFitness score	−0.070	0.235	−0.127	0.420	0.286	0.338	0.494	0.030	0.195	−0.104
ΔVFA [cm^2^]	−0.060	−0.192	0.085	−0.225	−0.266	−0.098	−0.142	−0.039	−0.252	0.104
ΔBody mass [kg]	0.029	−0.083	−0.096	−0.166	−0.241	−0.050	−0.097	0.110	−0.224	−0.066
ΔSMM [kg]	0.060	0.073	−0.232	0.023	−0.120	0.067	0.115	0.173	−0.167	−0.171
ΔBFM [kg]	0.006	−0.183	0.015	−0.267	−0.286	−0.123	−0.229	0.045	−0.215	0.029
ΔPBF [%]	−0.100	−0.184	0.066	−0.147	−0.272	0.055	−0.056	−0.021	−0.166	0.088
ΔECW [%]	0.061	0.074	−0.231	0.020	−0.122	0.064	0.113	0.178	−0.163	−0.169
ΔICW [%]	0.036	0.075	−0.205	−0.023	−0.123	0.051	0.077	0.160	−0.221	−0.195
ΔTBW [kg]	0.052	0.075	−0.222	0.003	−0.123	0.059	0.099	0.171	−0.186	−0.179

VFA, visceral fat area, BFM, body fat mass, SMM, skeletal muscle mass, BFM, body fat mass, PBF, percent body fat, ICW, intracellular water, ECW, extracellular water, TBW, total body water, HRrest, heart rate at rest, SBPrest, systolic blood pressure at rest DBPrest, diastolic blood pressure at rest, MET, metabolic equivalent, VO_2_ peak, peak oxygen consumption, HRpeak, peak heart rate, SBPpeak, peak systolic blood pressure, DBPpeak, diastolic blood pressure.

**Table 7:** Correlation of the BMI value with the results of the exercise test.

Variable	Δ HRrest [beats/min]	Δ SBPrest [mmHg]	Δ DBPrest [mmHg]	Δ Time [min]	Δ MET	Δ VO_2peak_ [ml/kg/min]	Δ Distance [m]	Δ HRpeak [beats/min]	Δ SBPpeak [mmHg]	Δ DBPpeak [mmHg]
ΔBMI [kg/m^2^]	0.077	−0.149	−0.130	0.439	0.626	0.222	0.442	−0.241	−0.241	0.098

BMI, body mass index; HRrest, heart rate at rest; SBPrest, systolic blood pressure at rest; DBPrest, diastolic blood pressure at rest; MET, metabolic equivalent; VO_2_ peak, peak oxygen consumption; HRpeak, peak heart rate; SBPpeak, peak systolic blood pressure; DBPpeak, diastolic blood pressure.

**Table 8:** Correlation of body mass index values with results of the exercise test.

Variable	Δ HRrest [beats/min]	Δ SBPrest [mmHg]	Δ DBPrest [mmHg]	Δ Time [min]	Δ MET	Δ VO_2max_ [ml/kg/min	Δ Distance [m]	Δ HRmax [beats/min]	Δ SBPmax [mmHg]	Δ DBPmax [mmHg]
ΔFitness score	−0.093	−0.105	0.144	−0.355	−0.392	−0.158	−0.347	−0.091	−0.078	−0.062
Δ VFA [cm^2^]	0.160	0.054	−0.097	0.320	0.548	0.174	0.310	−0.164	−0.091	0.085
Δ Body mass [kg]	0.163	−0.079	−0.120	0.178	0.528	0.219	0.217	−0.210	−0.226	0.003
Δ SMM [kg]	0.151	−0.143	−0.074	−0.125	0.317	0.155	−0.052	−0.284	−0.289	−0.090
Δ BFM [kg]	0.142	−0.039	−0.134	0.316	0.566	0.223	0.332	−0.148	−0.164	0.051
Δ PBF [%]	0.078	−0.149	−0.130	0.440	0.626	0.222	0.442	−0.241	−0.241	0.098
Δ ECW [%]	0.152	−0.143	−0.074	−0.125	0.315	0.155	−0.054	−0.281	−0.288	−0.088
Δ ICW [%]	0.148	−0.140	−0.034	−0.105	0.368	0.159	−0.025	−0.272	−0.296	−0.073
Δ TBW [kg]	0.151	−0.142	−0.059	−0.118	0.335	0.157	−0.043	−0.279	−0.292	−0.083

VFA, visceral fat area; BFM, body fat mass, SMM, skeletal muscle mass; BFM, body fat mass; PBF, percent body fat; ICW, intracellular water; ECW, extracellular water; TBW, total body water; HRrest, heart rate at rest; SBPrest, systolic blood pressure at rest; DBPrest, diastolic blood pressure at rest; MET, metabolic equivalent; VO_2_ peak, peak oxygen consumption; HRpeak, peak heart rate; SBPpeak, peak systolic blood pressure; DBPpeak, diastolic blood pressure.

## Discussion

The study results confirmed the effectiveness of both walking forms. However, more statistically significant differences were observed in the improvement of exercise tolerance, assessed by the exercise test, in the Nordic Walking group. A similar effect was found in the body composition analysis, but it did not reach statistical significance. A detailed description of the results of individual tests is presented below.

### Electrocardiographic exercise test

Assessment of exercise tolerance is an essential element of physical exercise intensity programming (17, 18). Its result is very important in qualifying for various training programs, including rehabilitation. The popularity of Nordic walking increased the interest in this form of activity among physiologists, biomechanics and physiotherapists (19). During exercise, stimulation of the sympathetic nervous system causes an increased oxygen demand of the heart muscle. The increase in heart rate, its contractility and systolic blood pressure make the ECG stress test an accurate diagnostic test.

Own research showed the effectiveness of both forms of training, but with regard to the level of exercise tolerance, assessed with the exercise test on a treadmill, more favorable results were observed in the Nordic walking group. It was probably influenced by the level of training intensity. In the group of women walking with poles, the energy expenditure associated with the form of training used was significantly higher than in the group without poles, because the intensive work of the upper limbs was additionally involved. Physiological changes in the body related to greater physical effort produced an effect similar to that in competitive or recreational sports, but of course at a much lower level. Comparing the results of the preliminary and final tests in terms of the magnitude of changes in the analyzed variables (deltas) of both studied groups, significant changes in favor of Nordic walking were noted in the duration of the exercise test (respectively: 1.13 vs. 0.63 min; *p* = 0.002), maximum oxygen consumption VO_2_max (respectively: 4.37 vs. 2.33 ml; *p* = 0.035) and the distance covered on the treadmill (respectively: 59.71 vs. 39.34 m; *p* = 0.045). With regard to the other indicators of the exercise test (MET, resting and peak heart rate, resting and peak blood pressure), favorable changes were obtained, but they were not statistically significant.

Kukkonen-Harjula et al. (12) investigated the effectiveness of walking and Nordic walking among women aged 50–60, leading a sedentary lifestyle but with a normal BMI index. After the end of the 13-week training cycle (classes were conducted 4 times a week for 40 min), they showed an increase in the maximum oxygen consumption (VO_2_max) in both groups (in the Nordic walking group by 2.5 ml/min/kg, in the group without poles by 2.6 ml/min/kg). In both groups, a reduction in the resting value and the exercise rate of heart contractions was also achieved. On the other hand, Mikalački et al. (20), examining the efficiency of older women (58.5 ± 6.90 years) participating in 3-month walking training with poles (3 times a week), obtained a result that also confirms its effectiveness. Based on the exercise tests performed before and after the training program, they observed a reduction in resting heart rate (84.93 vs. 73.42 bpm, *p* = 0.001), systolic and diastolic blood pressure (129.83/84.66 vs. 118.42/79.04 mmHg, *p* = 0.000) and increase in VO_2_max (15.75 vs. 21.83 ml/kg/min, *p* = 0.000). The high effectiveness of walking training with poles was also confirmed in the research by Tschentscher et al. (21) and Fritschi et al. (10), achieving a similar effect. It is also worth mentioning the first studies assessing the effectiveness of walking and Nordic walking among women. They were conducted in 1992 at the University of Wisconsin-La Crosse in the United States. The training lasted 3 months (4 times a week for 30–45 min). In both studied groups, an increase in the level of VO_2_max was observed, but the largest (8%) was in the Nordic walking group.

### Body weight composition

In both groups there was a significant increase in the percentage of adipose tissue in the body and the percentage of intracellular water, and a significant decrease in the percentage of extracellular water. There was also an increase in skeletal muscle mass in the Nordic walking group. With regard to the other components, the changes turned out to be insignificant, and in some cases, such as visceral obesity, the expected training effect turned out to be the opposite. The only explanation for such surprising results may be the lack of discipline associated with the diet during the entire training program. Before starting the training cycle, all the women were instructed about the dietary recommendations, indications and contraindications for eating certain products, the time of eating meals and their volume. During an interview conducted after the end of the program, some women admitted that they did not always follow the recommended diet. Perhaps the obtained results were influenced by the observation time, which in turn would be confirmed by the Willmore study (22). He performed a meta-analysis of 53 studies on changes in body mass composition under the influence of physical training with and without changing the diet. He found that the 6-month period of increased physical activity caused a decrease in body weight by an average of 1.6 kg, a decrease in fat mass by an average of 2.6 kg and an increase in lean mass by 1.0 kg. In studies with a shorter follow-up period of up to 12 weeks, no or insignificant changes were observed. A similar meta-analysis was conducted by Wing (23). An analysis of 29 randomized trials in which obesity was treated with only a low-calorie diet, or only increased physical activity, or a combination of these, indicated that the combined use of diet and increased physical activity resulted in the greatest (beneficial) changes in body weight composition. Grigolett et al. (24) when assessing the effectiveness of NW training in relation to anthropological parameters and body composition, also found high effectiveness of this form of training. Comparing the body weight composition of physically active and inactive women, aged >50 years, they found significant differences, more favorable in the group of active women, in terms of the percentage of cellular mass, percentage of body water, percentage of adipose tissue mass, percentage of lean tissue mass and muscle mass expressed in kilograms and as a percentage, as well as basal metabolic rate. Moreover, they observed significant differences in the percentage of total body water, percentage of lean mass, and percentage of adipose tissue mass. Bullo et al. (25) carried out a meta-analysis of the influence of Nordic walking on the physical fitness, body composition and quality of life of the elderly, based on 15 studies. They observed favorable changes after the end of the training program of seniors. They concluded that Nordic walking can be considered a safe and accessible form of aerobic exercise for the elderly, capable of improving cardiovascular outcomes, muscle strength, body composition, balance ability and quality of life. Analyzing the effects of NW training in the treatment of overweight over 3 and 6 months from the start of training Muollo et al. also found a significant reduction in adipose tissue, BMI and aerobic capacity (26).

### Correlation of the level of exercise tolerance and body mass components

The study additionally analyzed the correlation of changes (delta) in the results of body mass composition with changes (delta) in the stress test indices for both studied groups. In the case of the walking group, no significant correlation was found, while in the Nordic walking group, a significant positive relationship was found between the BMI index and the increase in the duration of the exercise test, the metabolic cost of MET and the maximum oxygen consumption. In addition, there was a significant correlation between changes (delta) in body fat percentage, body fat mass, body weight, visceral obesity and fitness score with changes (delta) in metabolic cost associated with exercise testing, changes (delta) in body fat percentage, and the increase in the duration of the exercise test as well as the percentage changes (delta) in the content of adipose tissue and the increase in the distance covered during the test. Obtaining so many relationships between changes in body mass composition and changes in exercise test indicators in the Nordic walking group may result from the greater intensity of the form of training used than in the normal walking group, which in turn contributed to the improvement of exercise tolerance itself. Although in both analyzed groups a similar significant change was found in relation to the same body composition parameters (percentage of adipose tissue, external and intracellular water), the intensity of the training played a key role. Moreover, if we analyze the results in both studied groups, we find that the dynamics of changes in the body mass composition, despite the lack of statistical significance, was higher in the Nordic walking group than in the normal walking group. Therefore, it can be assumed that, as mentioned earlier, the training intensity had an influence on the obtained result of the studied dependencies.

To sum up, the studies conducted in the group of women >50 years of age confirmed the effectiveness of walking training, regardless of whether it was with or without poles. Both forms of activity had a similar effect on the improvement of exercise tolerance, lipid profile and body weight composition. When analyzing changes in body mass composition in relation to changes in exercise tolerance, the Nordic walking group achieved a more favorable result. Therefore, it seems that this form of activity would be the most appropriate for women over 50. However, it must be remembered that when recommending Nordic walking training, due to its specific nature, one should always take into account the indications and contraindications. Regardless of the results of the experiment, walking with or without poles should be recommended as a safe and widely accessible form of physical activity, especially in relation to the elderly. A properly prepared and conducted training program based on the assessment of exercise tolerance is a very important element of both secondary and primary prevention.

## Study limitations

The main limitation of the study was the relatively short observation period, with limited frequency and duration of training. It resulted from the program framework (schedule of classes) imposed by the University of the Third Age. As shown by the results obtained in other studies, this period was too short. Another limitation of the study was that the number of participants was too small (group I – 32 people, group II – 34 people), which could also have an impact on the statistical analysis of the results. One more and perhaps the most important factor influencing the course of the experiment should also be mentioned. Since all women were hospitalized before and after training in order to perform appropriate diagnostic tests, in order not to interfere with work in the wards, the number of participants had to be limited to the necessary minimum.

## Conclusions

In both studied groups, a similar level of significant changes in the same body mass indices (percentage of adipose tissue, percentage of intra- and extracellular water) was obtained. In the other components, the results were not significant.

A relationship between the change in the level of exercise tolerance and changes in the body mass composition was observed only in the Nordic walking group.

## Data Availability

The raw data supporting the conclusions of this article will be made available by the authors, without undue reservation.
